# The data and characteristics of the human milk banks in mainland China

**DOI:** 10.1007/s12519-019-00226-6

**Published:** 2019-02-22

**Authors:** Xi-Hong Liu, Shu-Ping Han, Qiu-Fen Wei, Feng-Ying Zheng, Ting Zhang, Hui-Min Chen, Meng Mao, National Human Milk Bank Group, Children Health of the Chinese Medical Doctors Association, Xi-Hong Liu, Shu-Ping Han, Qiu-Fen Wei, Feng-Ying Zheng, Ting Zhang, Hui-Min Chen, Group of Child Health Care, Society of Pediatrics, Chinese Medical Association, Meng Mao, Xi-Hong Liu

**Affiliations:** 1 Clinical Nutrition Department of Guangzhou Women and Children Medical Center, Guangzhou, 510623 China; 2 Nanjing Maternal and Child Health Center Affiliated of Nanjing Medical University, Nanjing, China; 3 Guangxi Maternal and Child Health Hospital, Nanning, China; 4 The 4th People’s Hospital of Shaanxi Province, Xi’an, China; 5 Shanghai Children’s Hospital, Shanghai, China; 6 West China Second Hospital of Sichuan University, Chengdu, China

**Keywords:** Breast milk, Donor human milk, Human milk banking, Recipients

## Abstract

**Background:**

Human milk banks (HMB) have been established for over 100 years in North America and Europe. This study aimed to describe and summarize the operation and characteristics of the HMBs in mainland China since the first nonprofit HMB operated in 2013.

**Methods:**

Operation of HMB in mainland China is based on the standards and guidelines of the Human Milk Banking Association of North America and some countries in Europe and was modified to meet the needs and circumstances in China such as donation only in the local HMB by medical staff. We reviewed the descriptive data of these 14 HMBs and the clinical characteristics of recipients, the eligible milk donors and the donor milk retrospectively.

**Results:**

In mainland China, from March 2013 to December 2016, 14 nonprofit HMBs were developed and operational in public hospitals except one and located in the south, east, north and northwest of mainland China. In total, 2680 eligible donors donated 4608.2 L of breast milk. The mean age of these donors was 29.4 years with 60.6% receiving college education and 90.6% term delivery. A total of 4678 recipients including preterm infants (*n *= 2990, 63.9%), feeding intolerance (*n *= 711, 15.2%), maternal illness (*n *= 345, 7.4%), serious infection (*n *= 314, 6.7%), necrotising enterocolitis (*n *= 244, 5.2%), post-surgery (*n *= 38, 0.8%) and others (*n *= 36, 0.8%). The rate of discarded raw milk was only 4.4% because of hepatitis B and C or cytomegalovirus positivity.

**Conclusions:**

HMB has been developing rapidly in mainland China. Donor human milk was used not only for preterm infants but also for other ill children. But the sustainability of milk banking needs proper management and more financial support by relative health authorities and the government.

## Introduction

The first human milk bank (HMB) was established in Austria over a century ago [1]. Today, there are 233 active milk banks and 14 planned milk banks in Europe and 27 active milk banks in the United States, providing donor milk for ill and preterm infants [2, 3]. This growth has been stimulated by evidence showing the clinical benefits of breast milk or donor human milk (DHM) for preterm infants. Guidelines or recommendations for the operation of milk banking were created by the Human Milk Banking Association of North America (HMBANA) [4] and some European countries such as Italian and Britain as well as Australian [5–7]. The Committee on Nutrition of the European Society for Pediatric Gastroenterology, Hepatology, and Nutrition recommends that fresh own mother’s milk (OMM) is the first choice for preterm babies in neonatal intensive care units (NICUs), and then DHM should be the second choice [8]. The benefits of human milk have been well documented worldwide, especially to the preterm infants, such as decreasing rates of sepsis, necrotizing enterocolitis, allergies and sudden infant death syndrome, while increasing IQ, tolerance for feeding and long term developmental, neurocognitive and cardiovascular outcomes [1, 8–11].

HMB was a novel concept in mainland China when the first HMB opened in March, 2013 in Guangzhou which is one of the largest open cities in southern China. Subsequently human milk banking developed very fast. Until December 2016, there were other 13 ones operated in succession which were located in big cities in the south, east, north, central and southeast of China, respectively. DHM was used not only for preterm infants but also for other ill children. This article is a description of human milk banking practices and experiences with donor screening and processing, as well as the use of DHM in mainland China.

## Methods

The human milk banking standards of practice in mainland China referred to the guidelines published by HMBANA and some European countries but with Chinese characteristics. All donors are screened through a questionnaire survey for health history first, and then signed an informed consent form stating that they donated their breast milk voluntarily and promised not to interfere in the use and distribution of the donor milk. The donor inclusion criteria were strict as below: (1) in good physical and psychological health; (2) no tobacco, illegal drug, or excessive alcohol use; (3) not regularly using medication or herbal supplements; (4) have not received organ or tissue transplant or blood transfusion in the past 12 months; (5) mother of a healthy growing infant without any congenital diseases or genetic diseases; (6) promising not to deprive her infant’s nutrition and only to donate the extra breast milk. At last, the mothers should undergo blood tests for human immunodeficiency virus, hepatitis B and C, cytomegalovirus, and syphilis. All tests were undertaken in laboratories of respective hospital. After the questionnaire and the results of blood tests were confirmed by the doctors in milk banks, the qualified donors would be informed by telephone and could come to the milk banks and donate their milk freely. These screening criteria met international recommendations for human milk banking [5–8]. Every 3 months the blood tests were repeated and at the same time the donors’ general health status were checked regularly until the 10th month postpartum. At the beginning of practice, all the donors were required to donate their milk at each milk bank where it was collected only by the medical staff with a hospital grade sterilized One-Day Pump. Before the formal national policy was published, frozen milk collected at home by the donor themselves was not accepted to ensure the safety of DHM.

The raw donor milk was pooled from the same donor and separated into several containers then stored in refrigerator at 4 °C immediately. Before pasteurization, one sample was randomly selected from each donor for bacteriological examination. If the total bacterial colony counts were > 10^5^, the milk was considered to be “unqualified” or “pathological” and would be discarded. The raw milk was typically pasteurized by Holder pasteurization (62.5 °C for 30 minutes) according to the recommendation by HMBANA and EMBA. At the beginning, we tested each sample for bacteriological detection and after pasteurization only some samples were tested randomly from each batch. Pasteurized milk was discarded if any microbial content was found.

The recipients were the inpatient preterm infants or those of very low birth weight, as well as infants with necrotizing enterocolitis. In some hospitals, the recipient criteria expanded to other conditions determined at the clinical level as below: (1) immunologic deficiency caused by transplantation, radiotherapy or chemotherapy; (2) requiring nutritional support after a major operation (such as congenital heart disease, necrotizing enterocolitis, intestinal atresia); (3) serious infectious diseases such as sepsis or severe pneumonia; (4) cow’s milk protein allergy with malnutrition, etc.

Each HMB was approved by the hospital’s ethics committee before it was established. The information and data of each HMB was recorded and reviewed including the donors, the recipients, the process and use of DHM. We used descriptive statistics by Microsoft Excel.

## Results

### The data and running trend of milk banks in mainland China

**Fig. 1:**
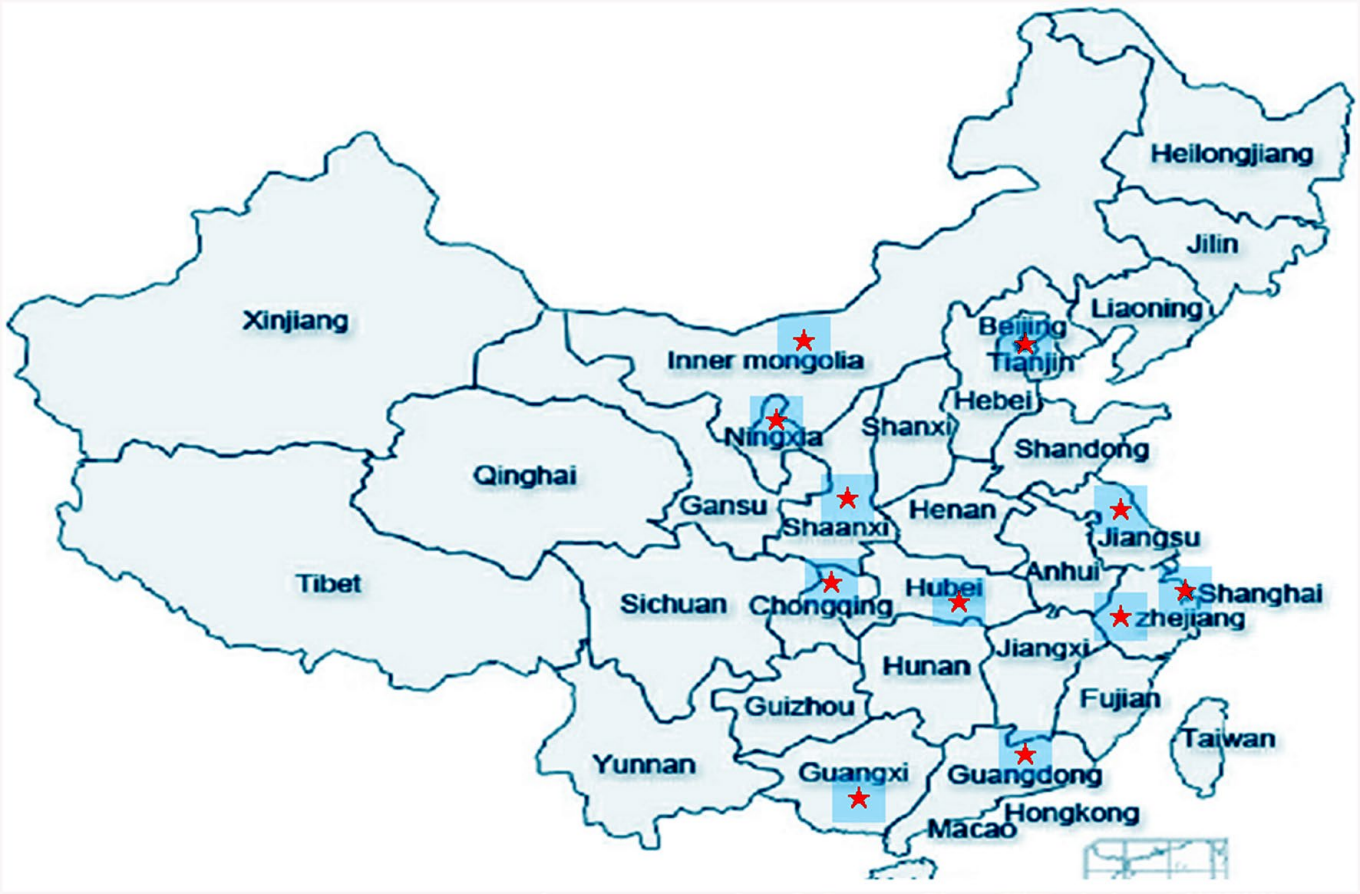
The map of HMBs in mainland China. There were three HMBs in Guangdong, and two in Jiangsu. *HMB* human milk bank

During the past 3.5 years from March 2013 to December 2016, there were 14 HMB developed and operated successfully in mainland China (Fig. 1, Table 1). The total donors increased over the years from 309 in 2013 to 1429 in 2016. Similarly, the total donation times and donated milk volumes increased several times during these 3 years, from 1273 to 8331 and from 230.9 to 2740.7 L, respectively (Table 1).

**Table 1:** Data of milk banking in mainland China (2013–2016)

Variables	2013	2014	2015	2016
Number of human milk banks	2	4	9	14
Total eligible donors	309	279	663	1429
Total donation times	1273	2330	2933	8331
Total donated milk volumes (L)	230.9	813.9	822.7	2740.7
Average volumes (L)/donor	0.75	2.92	1.24	1.92
Average times/donor	4.12	8.35	4.42	5.83
Average donors/bank/mon	23.8	8.5	12.2	13.6
Average times/bank/mon	53	127.6	81.1	89.1

Unfortunately, the average volumes (L) per donor, average times per donor, average donors per bank per month and average times per bank per month did not increased over the years (Table 1).

### The characteristics of the eligible donors

In total, there were 2680 eligible donors in these 14 milk banks during these 3.5 years in mainland China. Most of the donors delivered at full term (90.6%) and 81.8% of them were 25–35 years old. The lactating women who donated less than 3 times accounted for 45.0%, and only 24.4% donated more than 10 times. 77.2% of them began to donate milk after one month postpartum period, 60.6% of them had college education. Other characteristics of the donors such as the mode of delivery, the occupation and the way of getting donation information were analyzed below (Table 2). There were few non-eligible donors until now because of strict screening.

**Table 2:** Characteristics of the eligible donors in mainland China

Variables	*n* (%)
Donation times	
< 3	1206 (45.0)
3–9	820 (30.6)
≥ 10	654 (24.4)
First donation time (postpartum)	
About 1 wk	209 (7.8)
1 wk–1 mon	402 (15.0)
1–3 mon	847 (31.6)
3–6 mon	796 (29.7)
6–10 mon	426 (15.9)
Preterm/term delivery	
Preterm	252 (9.4)
Term	2428 (90.6)
Mode of delivery	
Vaginal delivery	1801 (67.2)
Cesarean delivery	879 (32.8)
Age (y)	
20–25	255 (9.5)
25–30	1313 (49.0)
30–35	879 (32.8)
> 35	233 (8.7)
Occupation	
Company employee	622 (23.2)
Government employee	169 (6.3)
Medical worker	204 (7.6)
Teacher	142 (5.3)
Self-employed	233 (8.7)
Housewife	407 (15.2)
Others	903 (33.7)
Education	
Above bachelor’s degree	236 (8.8)
Bachelor’s degree	1388 (51.8)
Junior college	662 (24.7)
Senior high school	255 (9.5)
Junior high school	139 (5.2)
Ways of getting milk donation information	
Internet media	871 (32.5)
Medical staff	788 (29.4)
Local television	399 (14.9)
Newspapers	271 (10.1)
Others	351 (13.1)

### The use of donated milk and the characteristics of the recipients

**Fig. 2:**
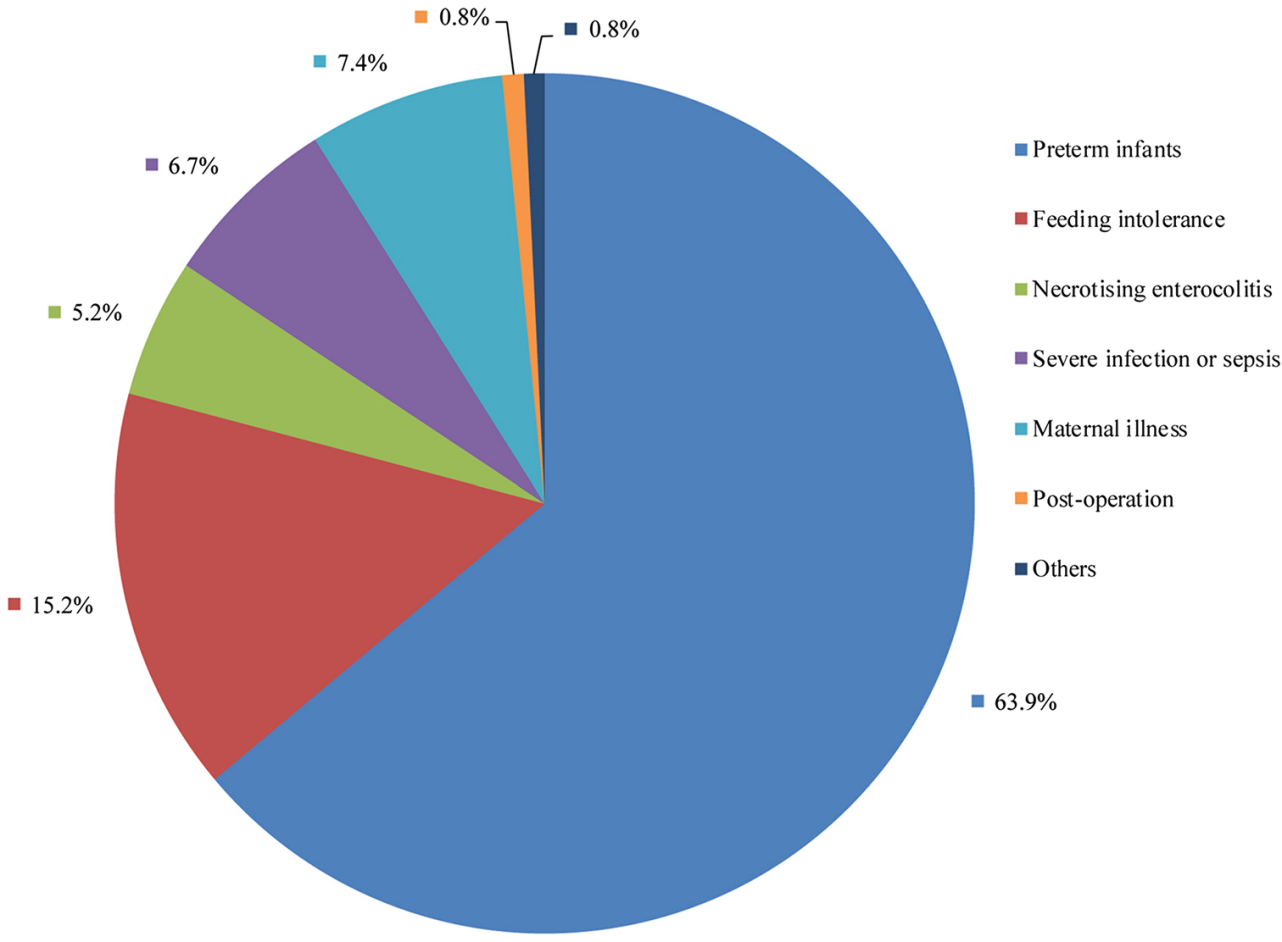
Disease types of the recipients

A total of 4678 received DHM as nutritional therapy due to several indications including prematurity (*n *= 2990, 63.9%), feeding intolerance (*n *= 711, 15.2%), maternal illness (*n *= 345, 7.4%), serious infection (*n *= 314, 6.7%), necrotising enterocolitis (*n *= 244, 5.2%), post-surgery (*n *= 38, 0.8%) and others (*n *= 36, 0.8%) (Fig. 2). Anyhow, most of the recipients were born before 37 weeks of gestation age and below 2500 g of birth weight. But, 79.0% of them only received the DHM less than 15 days, 18.1% for less than 1 month (Table 3).

**Table 3:** Characteristics of the recipients

Variables	*n* (%)
Gestation age of recipients (wk)	
< 28	217 (4.6)
≥ 28 – < 31	1316 (28.1)
≥ 31 – < 34	2273 (48.6)
≥ 34 – < 37	633 (13.5)
≥ 37	239 (5.1)
Birth weight of recipients (g)	
< 1000	226 (4.8)
≥ 1000 – < 1500	1572 (33.6)
≥ 1500 – < 2500	2355 (50.3)
≥ 2500	525 (11.2)
Duration of receiving donor human milk	
< 15 d	3696 (79.0)
≥ 15 d – < 1 mon	847 (18.1)
≥ 1 – < 2 mon	112 (2.4)
> 2 mon	23 (0.5)

## Discussion

Since the early 1990s, the Chinese Government has introduced policy of “Baby Friendly Hospital Initiative” together with regulations on the marketing of infant formula. As a result, the breastfeeding rate appeared to be increasing [12–14]. Unfortunately, the rate of exclusive breastfeeding declined rapidly soon afterwards. Many local studies showed the breastfeeding rate in China was far below the target of 80% “exclusive breastfeeding to 4 months” proposed by the Chinese Children’s Development Plan [15–17] and below the goal recommended by the World Health Organization (WHO) of “exclusive breastfeeding for 6 months” [18]. It was reported that only 59.4% had initiated breastfeeding early (i.e., within 1 hour of birth); only 55.5% and 9.4% had continued breastfeeding for 1 and 2 years, respectively, and only 28.7% of infants younger than 6 months had been exclusively breastfed [19]. And United Nations International Children’s Emergency Fund reported that in 2014 the rate of exclusive breastfeeding among infants aged 6 months in China was only 28% with no increase in 3 years [20]. Multiple factors affected breastfeeding rates, including demographics, social support, socioeconomic status, parents’ educational backgrounds, and high cesarean section rates [21, 22], etc. Meanwhile, many misconceptions about the breastfeeding still existed both in the general population and among healthcare professionals even in the neonatology department or NICU, such as the nutritional value of infant formula being the same as breast milk, breast milk not having any nutrition value after 10-month postpartum, and that DHM was not safe.

Theoretically, the benefits of breast milk feeding are well known like optimal nutrition, easy digestibility, and immunologic protection for infants, as well as improved nutritional, psychological, and cardiovascular health and cognitive abilities in later life [23–26]. In addition, human milk contains growth factors that can protect immature tissues, promote maturation, particularly of the gastrointestinal tract, and promote healing of damaged tissues. Since the introduction of DHM, many studies have demonstrated the clinical efficacy for preterm infants [8, 9, 27]. At the same time, the benefits of human milk for nourishing preterm infants have been demonstrated unequivocally in recent decades. It was very clear that the first recipients of DHM were preterm infants, especially the very and extremely low birth weight infants.

Occasionally, DHM has also been used to treat illnesses in older children or even in adults, such as cystic fibrosis, failure to thrive, congenital anomalies, necrotizing enterocolitis, immune deficiency, cardiac postoperative therapy, and cancer [28–32]. Similarly, short-term clinical and lifesaving effects of DHM in other seriously ill infants and children were proven, especially in children with postoperative intestinal problems, which current nutritional knowledge could not explained easily [33]. Obviously, future studies should address the mechanisms for these benefits.

As shown above, the operation of human milk banking in mainland China was over 100 years later than the first HMB in the world. But from the speed of development in the past 4 years, it was over 10 times faster (3 HMBs in 10 years and 14 in 4 years). The total number of HMBs and the donors, the total volume of DHM and the number of recipients increased rapidly and steadily these years, but the average number of donors of each milk bank per month did not increased accordingly. Each HMB had an average of only 12–13 new donors per month, and the average volume of DHM was less than 1000 mL per day which was far from enough for the need of hospitalized children. Why? The reasons were manifold. First, the breast milk feeding rate was still very low in China as explained above. Second, all donors were encouraged to donate their human milk at each HMB with the help of trained nurse. Frozen milk collected at home was refused generally. Therefore, the number of donors, and the total volume of DHM were both limited obviously. In the future, lactating women’s knowledge and attitude about milk donation should be promoted several educational channels.

Most eligible milk donors were well-educated (bachelor degree or above accounted for 60.6%) and employed women. Approximately 90% of the donors had full-term delivery; most of their age was between 25 and 35 years old. These features were similar to the Taiwan HMBs’ [34]. However, most of the donors only donated less than 3 times, the donors who could continue donation more than 10 times were less than 1/4. 77.2% of the donors began to donate their milk after at least 1 month postpartum because of the traditional Chinese custom of sitting on the moon. Unfortunately, the donors also significantly reduced after 6 months postpartum. Of course, the data were consistent with the decline in breastfeeding rate after the end of maternity leave in China. In general, both the number of donors and the number or the amount of donations was too limited and dissatisfactory. Therefore, the future efforts should be focusing on increasing milk donation and promoting the benefits of breastfeeding. At the same time, the donation process should be changed to offer more convenience to those intended donors, such as: (1) drive mobile collection carts into community; (2) offer some kind of benefits to donors such as free parking space in the hospital, payment of the bus or taxi to the donor from home to the bank and back, free check-up visit to their infants, a small toy-gift for their babies, etc.; (3) organize collection points in different areas of the city such as pharmacies; and (4) invite more volunteers to serve at the donors’ home.

OMM is the first choice in preterm infant feeding. When OMM is not available or insufficient, DHM is the recommended alternative. When neither OMM nor DHM is available, preterm formula should be used. This is the guideline of the European Society for Pediatric Gastroenterology, Hepatology and Nutrition and also is the preterm infants’ feeding recommendation of WHO [8, 35]. In mainland China, hospitalized preterm infants were prioritized in DHM distribution. 94.8% of the recipients were premature babies, and most of their gestational age was 28–34 weeks, and majority (88.8%) were low birth weight infants, including extremely low birth weight infant and very low birth weight infant. Among these preterm infant recipients, most received DHM within 24 hours after birth, which was similar to the situation of DHM’s clinical application in European and American countries [11, 36, 37].

Due to the difference in time of operation, the number of donors, the donor milk volume and the number of recipients was quite different between each HMB in mainland China. However, the average DHM consumption per recipient varied from 313 to 5794 mL, and the duration receiving DHM varied from 4.4 to 35 days, most of which was not more than 15 days (79%). The reason for such short duration of DHM consumption were as follows: (1) some recipients had relative big gestational age or ideal birth weight whose conditions were stable and only stayed in hospital for several days; (2) there was not enough DHM for all preterm infants and aim was to provide DHM for these babies within the first few days after admission to achieve feed tolerance as soon as possible; (3) some recipients’ mothers had their own breast milk to feed their babies.

In Taiwan, they kept supplying DHM to infants in need even after discharge [34]. However, the policy was quite different from other milk banks in Europe and USA. In many countries, DHM was only provided to the sick infants in hospital or until gastrointestinal tolerance for 2 weeks. The benefits or effect of human milk or DHM on morbidity, specifically necrotising enterocolitis, late onset sepsis, retinopathy of prematurity, bronchopulmonary dysplasia, early achievement of full feeding, encourage successful catch-up growth and neurodevelopment in preterm infants have been well known [9–11, 36, 37]. Meanwhile, some studies found that these protective effects showed a time- and dose-dependent relationship [38]. Therefore, it was still necessary to observe and verify whether there was significant effect on these premature babies when they were provided with such a short time and so little HDM.

In mainland China, DHM was also provided to other sick children, such as those with feeding intolerance, severe infection as severe pneumonia or sepsis, malnutrition after major surgery, leukemia after chemotherapy, graft versus host response after transplantation, Pierre-Robin syndrome, or other situations including lack of breastfeeding because of mother’s illness. And the clinical effects were obvious, especially for that acute malnutrition caused by infection or surgery [33]. Actually, DHM was reported earlier to be used on other patients, even adult patients [38]. There was a study which showed that DHM improved the quality of life measures in the physical, psychological, and spiritual domains for most cancer patients [25]. DHM may be a lifesaving therapy for infants and young children with unusual medical conditions. In 2002, more than 300 infants and young children and 15 adults received donor milk from 6 milk banks in the United States and 1 milk bank in Canada [39].

Nevertheless, the human milk banking developed rapidly in mainland China, there were following some issues needing to be solved and improved which were also the limitation of this study: (1) the standards of operation of human milk banking should be unified. There were half of these 14 HMBs only collected the fresh DHM in HMB by nurses, other 7 HMBs also collected frozen DHM; (2) there were big differences in the amount of milk donated and the number of donors between each HMB; (3) at present, both donation and use of DHM were totally free in mainland China, and there was no charge standard for the clinical application. Anyway, the cost of HMB was relatively high, and almost all cost of the human milk banking operation was undertaken by the local hospital. Meanwhile, some local public welfare organizations or groups set up special fund for human milk donation. But how long would this free donation plus free use pattern persist?

Clinically, the use of DHM has been shown to have many benefits. Therefore, the HMB should be considered a reasonable and effective tool in the delivery of health care to infants and children. National policy from the government or professional guidelines from academic societies should be introduced urgently to assure HMB going well that included the use of donor milk as an extension of policies related to the collection, storage, and use of own mothers’ milk. HMB should be an integral part of Chinese health care, especially as part of the “baby-friendly hospital” policy as it was in other countries, and promoted, protected and supported by government.
